# mTORC2: a multifaceted regulator of autophagy

**DOI:** 10.1186/s12964-022-00859-7

**Published:** 2023-01-05

**Authors:** Yanan Sun, Huihui Wang, Taiqi Qu, Junjie Luo, Peng An, Fazheng Ren, Yongting Luo, Yixuan Li

**Affiliations:** 1 Key Laboratory of Precision Nutrition and Food Quality, Department of Nutrition and Health, China Agricultural University, Beijing, 100083 China; 2 College of Food Science and Engineering, Gansu Agricultural University, Lanzhou, 730070 China

**Keywords:** Autophagy, mTORC2, AKT, PKC, SGK-1

## Abstract

**Abstract:**

Autophagy is a multi-step catabolic process that delivers cellular components to lysosomes for degradation and recycling. The dysregulation of this precisely controlled process disrupts cellular homeostasis and leads to many pathophysiological conditions. The mechanistic target of rapamycin (mTOR) is a central nutrient sensor that integrates growth signals with anabolism to fulfil biosynthetic and bioenergetic requirements. mTOR nucleates two distinct evolutionarily conserved complexes (mTORC1 and mTORC2). However, only mTORC1 is acutely inhibited by rapamycin. Consequently, mTORC1 is a well characterized regulator of autophagy. While less is known about mTORC2, the availability of acute small molecule inhibitors and multiple genetic models has led to increased understanding about the role of mTORC2 in autophagy. Emerging evidence suggests that the regulation of mTORC2 in autophagy is mainly through its downstream effector proteins, and is variable under different conditions and cellular contexts. Here, we review recent advances that describe a role for mTORC2 in this catabolic process, and propose that mTORC2 could be a potential clinical target for the treatment of autophagy-related diseases.

**Video abstract**

**Graphical abstract:**

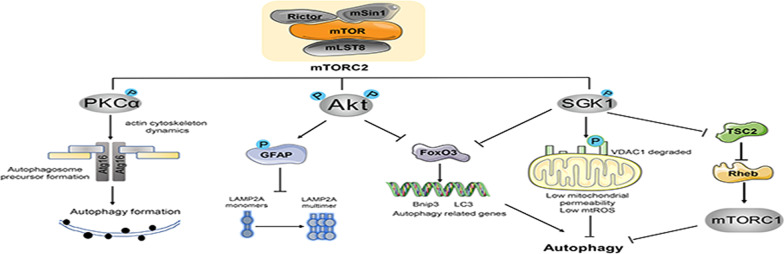

**Supplementary Information:**

The online version contains supplementary material available at 10.1186/s12964-022-00859-7.

## The importance of autophagy in cell homeostasis

**Fig. 1:**
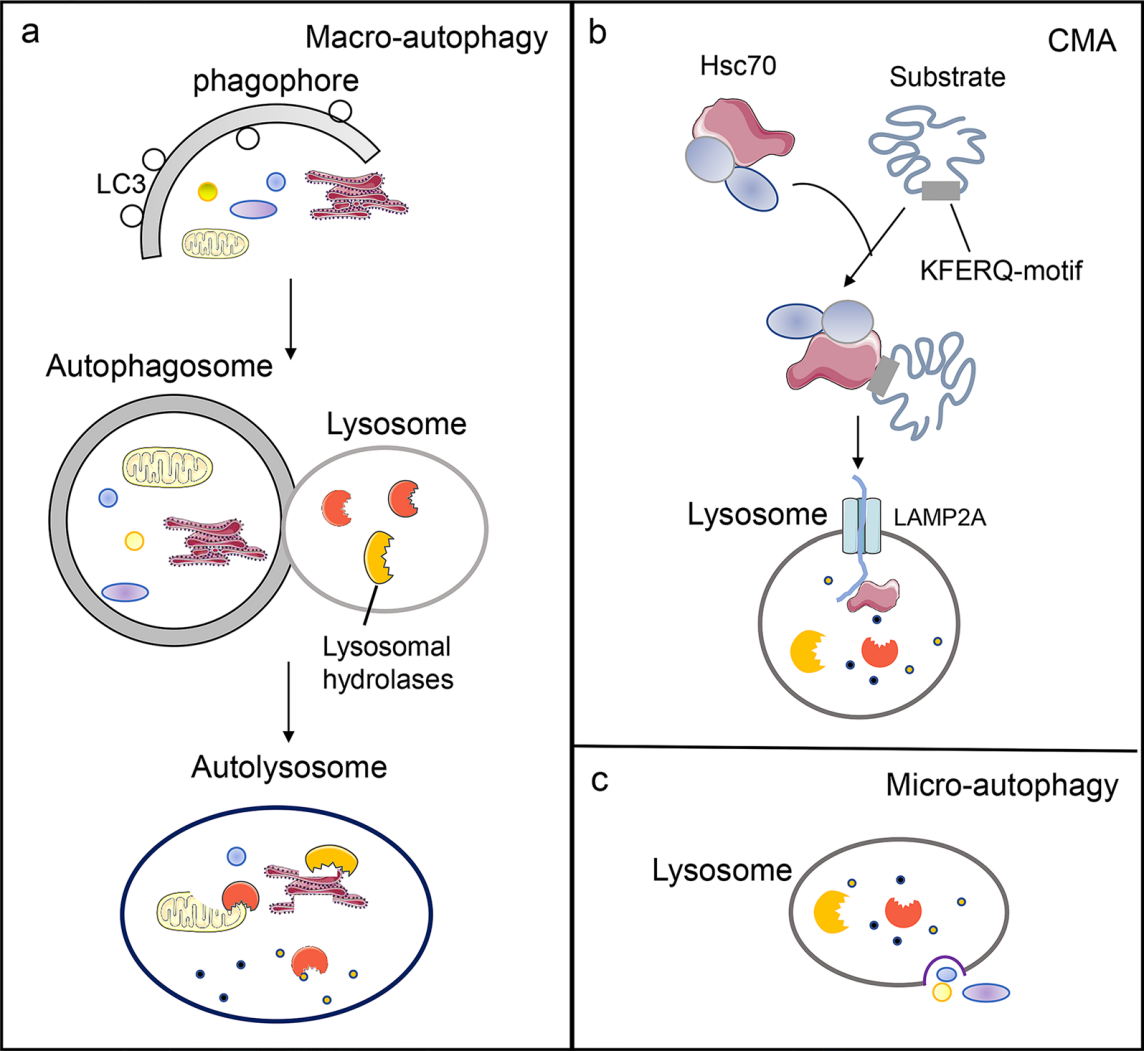
Three major subtypes of autophagy. **a** Macroautophagy is characterized by the de novo formation of autophagosomes and the fusion of autophagosomes and lysosomes. **b** CMA relies on the Hsc70 complex to identify and deliver substrate proteins to the lysosomes. **c** Microautophagy is characterized by the invagination or deformation of the lysosomal membrane for substrate entry

Autophagy and the ubiquitin proteasome system (UPS) are two major intracellular degradative mechanisms that mediate the catabolism of proteins and organelles. The proteasome system is responsible for the degradation of short-lived proteins and soluble unfolded/misfolded proteins and polypeptides, while the autophagy-lysosome system mediates the degradation of long-lived proteins, insoluble protein aggregates and damaged organelles [1, 2]. Autophagy can be subdivided into three subtypes, termed macroautophagy (Fig. 1a), chaperone-mediated autophagy (CMA) (Fig. 1b) and microautophagy (Fig. 1c), depending on the methods of substrate acquisition [3]. It is a highly conversed cellular degradation and recycling process in all eukaryotes that contributes to the maintenance and establishment of homeostasis in response to environmental and cellular stresses. Autophagy is comprised of several steps, including induction and nucleation, elongation, closure and maturation, fusion and degradation [4]. Dysfunction of this multi-step process results in the development of multiple human diseases, such as cancer, neurodegeneration and aging [5–7]. Thus, understanding the regulatory basis for autophagy holds the key to rethinking many fundamental pathophysiological processes, and knowing how to modulate this process may present novel therapeutic opportunities for the treatment of a broad range of autophagy-related diseases.

Macroautophagy is the most well characterized autophagic process. Upon induction of macroautophagy, autophagosome generation begins at a single perivacuolar site called the phagophore assembly site in yeast, and at multiple sites throughout the cytoplasm in mammalian cells. The biosynthesis of autophagosomes involves the appearance of a phagophore in the cytoplasm, which then expands, sequestering cytosomal components, before eventually sealing to form a spherical double-membraned vesicle called the autophagosome The newly-formed autophagosome then translocates to and fuses with the lysosome to form an autolysosome. Degradation of the cargo contained within the autophagosome then occurs inside the autolysosome [3]. In contrast, CMA is a highly selective autophagic process that requires a chaperone, the heat shock cognate 71 kDa protein (Hsc70), for target degradation. CMA selectively identifies its substrate proteins based on the presence of a sequence-specific pentapeptide on the substrate. During CMA, Hsc70 and other co-chaperones recognize the pentapeptide KFERQ on the substrate protein. The substrate protein is then delivered by Hsc70 to the lysosome membrane, where Hsc70 assists in substrate unfolding [8]. Once unfolded, the substrates bind to monomers of the lysosomal membrane receptor lysosome-associated membrane protein type 2A (LAMP2A), thereby promoting multimerization of LAMP2A [9]. The substrate proteins are translocated into the lysosomal lumen and degraded by lysosomal hydrolases. Microautophagy is a process whereby substrates enter lysosomes by invagination or deformation of the lysosomal membrane [4]. In general, these three types of autophagy maintain cellular homeostasis and survival.

In conclusion, the biological roles of autophagy are degrading intracellular components, such as misfolded proteins and damaged organelles, maintaining homeostasis in living organisms. Defective autophagy function is the cause of many diseases, such as various types of neurodegenerative diseases. The main pathology of neurodegenerative diseases, including Alzheimer’s diseases (AD), Parkinson diseases (PD), amyotrophic lateral sclerosis (ALS) and the polyglutamine (ploy Q) diseases, is a low level of autophagy and accumulation of misfolded proteins and damaged organelles in neurocyte [10]. Deregulation of autophagy is also involved in the pathogenesis of cancers. While the role of autophagy in cancer is controversial, which depends on the type and stage of cancers [11]. At the preliminary stage of cancer, autophagy can slow down the transformation of normal cells into tumor cells by protecting cells from ROS-induced damage to DNA and proteins [12]. At the late stages, autophagy has a tumor promotion effect through limiting DNA damage and supplies available nutrients [13]. So understanding the role of the autophagy in cancers is essential for cancer management. One of main characteristics aging is autophagy inhibition, thus accumulation of dysfunctional organelle, ROS and misfolded proteins in senescence cells. Previous studies suggested that autophagy was a positive longevity modulatory factor. For example, in mice activating autophagy by disruption of Beclin1-Bcl2 complex promotes longevity [14]. Although prevailing notion shown that autophagy is beneficial for longevity, otherwise under certain circumstances autophagy also play a detrimental role in health. Elevated autophagy and mPTP opening shorten lifespan [7]. Further work will be needed to detect the relationship between autophagy and aging. A better understand the interaction of mTORC2 plays in the complex process of autophagy will be great importance to autophagy-related diseases treatment.

## Composition and function of mTORC2

**Fig. 2:**
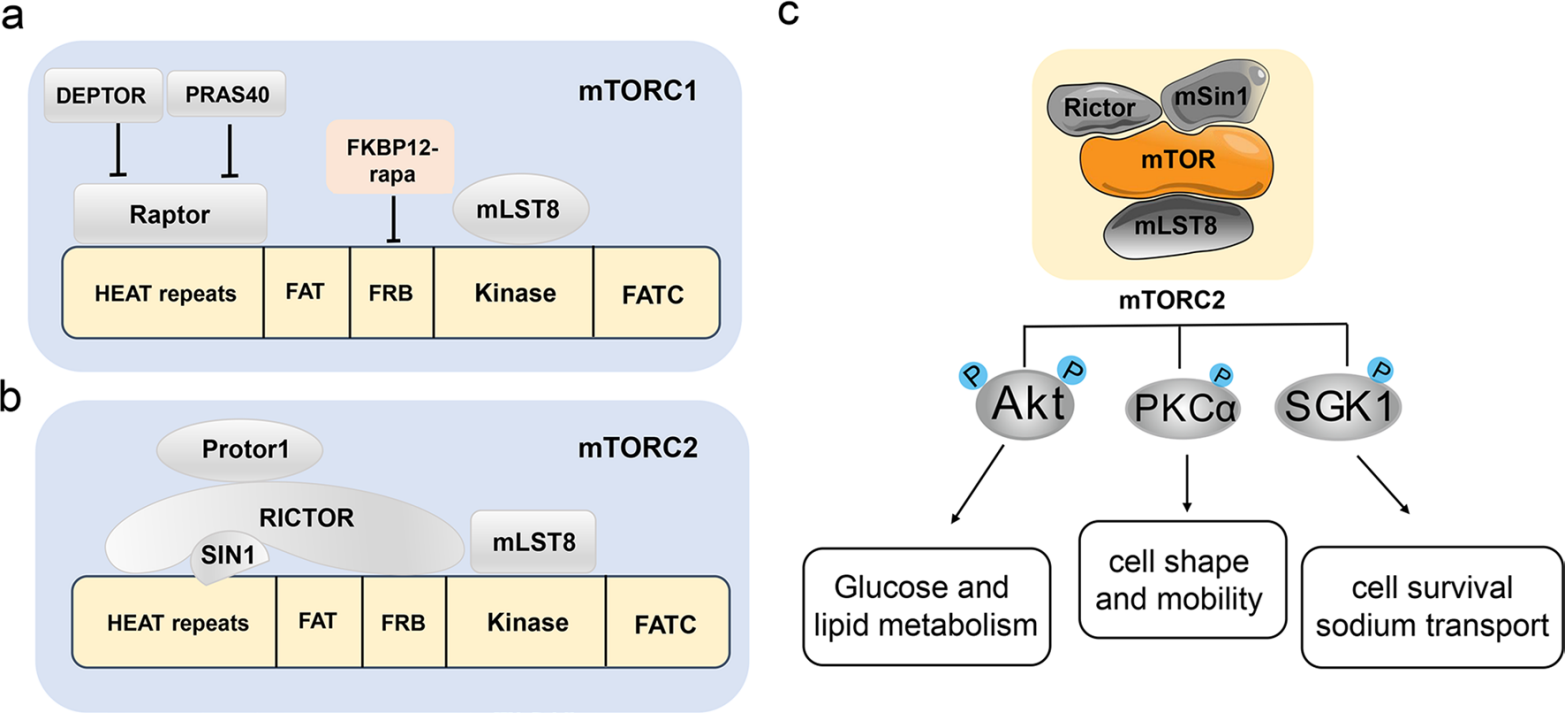
The composition and function of mTORC2. **a** The composition of mTORC1. **b** The composition of mTORC2. **c** mTORC2 exerts its various biological functions by phosphorylation of AGC kinases, including AKT, PKC and SGK-1

Mechanistic target of rapamycin (mTOR), an evolutionarily conserved serine/threonine protein kinase that belongs to the (phosphatidylinositol-3-kinase-related kinase) PI3K-related kinase family, coordinates eukaryotic cell growth and metabolism with environmental and intracellular inputs, such as nutrients, energy and growth factors [5]. mTOR functions through two structurally and functionally distinct complexes, mTORC1 and mTORC2 (Fig. 2a and b). While both mTORC1 and mTORC2 complexes contain the shared mTOR catalytic subunit, mammalian lethal with Sec13 protein 8 (mLST8) and DEP domain containing mTOR-interacting protein (DEPTOR), mTORC2 has two distinctive components, namely rapamycin-insensitive companion of mTOR (Rictor) and mammalian stress-activated protein kinase-interacting protein 1 (mSin1) [15–17]. Deletion of Rictor disrupts mTORC2 assembly and activity, suggesting that it has a profound impact on mTORC2 integrity and stability [18], while mSin1 is responsible for substrate recruitment and selection [19]. In addition, mLST8, which associates with the catalytic domain of mTOR and stabilizes the kinase activation loop, is essential for mTORC2 function, but not that of mTORC1 [20].

In mammalian cells, mTORC1 predominantly regulates mRNA translation, metabolism (de novo lipid synthesis, nucleotides synthesis and glycolysis) and protein turnover (autophagy and lysosomal biogenesis) through phosphorylation of its downstream effectors [21–24]. mTORC2 exerts its various biological functions, including cell proliferation, survival and cytoskeletal organization, through phosphorylation of AGC kinases, including (protein kinase B) AKT, (protein kinase C) PKC and (serum- and glucocorticoid-inducible kinase 1) SGK-1 (Fig. 2c) [23, 24]. AKT functions as a critical regulator of cell growth, proliferation, metabolism and cellular survival [25]. The SGK family is highly homologous to AKT, and shares similar upstream activators and downstream targets, that regulate cell growth, proliferation, survival and migration [26]. The phosphorylation of PKCs by mTORC2 plays an important role in regulating cell shape and mobility, as well as protein stability and solubility [27–29].

While mTORC1 is considered to be the main gateway to autophagy [15], the function and regulation of mTORC2 in autophagy remains poorly defined. However, emerging evidence indicates that mTORC2 also has an important role in autophagy, especially mitophagy (the selective degradation of mitochondria by autophagy), as well as a key positive modulator of longevity [7]. It is becoming clear that, unlike mTORC1, which negatively regulates autophagy, the role of mTORC2 in autophagy is more complicated and diverse. The role of mTORC2 in autophagy depends on its downstream effector proteins, cellular contexts, as well as distinct environmental stimuli. In this review, we will discuss the most revolutionary concepts regarding the regulatory mechanism of mTORC2 on autophagy, as well as its potential implications in autophagy-related disorders.

## mTORC2 regulates autophagy by activating its downstream effectors

### The mTORC2/AKT axis in autophagy

AKT is the most important and well characterized effector of mTORC2. Upon stimulation by growth factors, AKT is recruited to the plasma membrane through the interaction of its PH domain with PI3K-induced PIP3 [30]. PIP3 also triggers membrane recruitment of phosphoinositide-dependent protein kinase 1 (PDK1) and mTORC2, which phosphorylate T308 (in the activation loop) and S473 (in the hydrophobic motif) of AKT, leading to full activation of AKT [31, 32]. S473 is also phosphorylated by other kinases such as DNA-dependent protein kinase (DNA-PK) and integrin-linked kinase (ILK). However, the main activator of S473 kinase is thought to be mTORC2, since inactivation of mTORC2 results in a dramatic decrease in S473 phosphorylation [18, 32, 33].

AKT is the major downstream effector of insulin/PI3K-induced mTORC2 activity [18]. AKT regulates cell survival, growth, and proliferation through the phosphorylation and inhibition of several downstream targets, such as the metabolic regulator (glycogen synthase kinase-3β) GSK3β, transcription factor (forkhead box O1/3a) FOXO1/3a, and the mTORC1 inhibitor (tuberous sclerosis complex) TSC2 [5]. In skeletal muscle, the mTORC2-mediated effects on autophagy are dependent on the phosphorylation of AKT at the S473 residue [34]. Knockdown of RICTOR inactivates AKT, leading to the nuclear translocation of the transcription factor FOXO3, which is necessary for the induction of autophagy through the transcription of autophagy-related genes, including (microtubule-associated protein light chain 3) LC3 and Bnip3 [34]. Thus, mTORC2 acts as a negative regulator of autophagy through the AKT/FOXO3 signaling axis in skeletal muscle. Consistent with this study, miR-15a and miR-16 were shown to increase autophagic flux by directly targeting and downregulating Rictor, leading to the inactivation of AKT [35].

As well as canonical autophagy, mTORC2 is also essential for the regulation of CMA through AKT [36]. The binding of specific substrates to LAMP2A induces the formation of a multimeric complex, which is disassembled into monomeric forms of LAMP2A once the substrates cross the lysosomal membrane. Thus, the dynamics of LAMP2A play a critical role in the regulation of CMA [9]. Glial fibrillary acidic protein (GFAP) modulates the dynamics of LAMP-2A assembly and disassembly [37]. Dephosphorylated GFAP has a high binding affinity for LAMP2A, and binds to LAMP2A in its multimeric form, thereby contributing to its stabilization [37]. mTORC2 promotes the monomeric forms of LAMP2A through AKT-mediated phosphorylation of GFAP, resulting in low levels of CMA [36, 38]. In contrast, leucine-rich repeat protein phosphatase 1 (PHLPP1) deactivates AKT to promote dephosphorylation of GFAP, which then facilitates the formation of a LAMP2 multimer complex and high levels of CMA [36, 38]. mTORC2 and PHLPP1, therefore, act as endogenous CMA inhibitors and stimulators, respectively, and regulate CMA through the modulation of lysosomal AKT activity [36, 38].

**Fig. 3:**
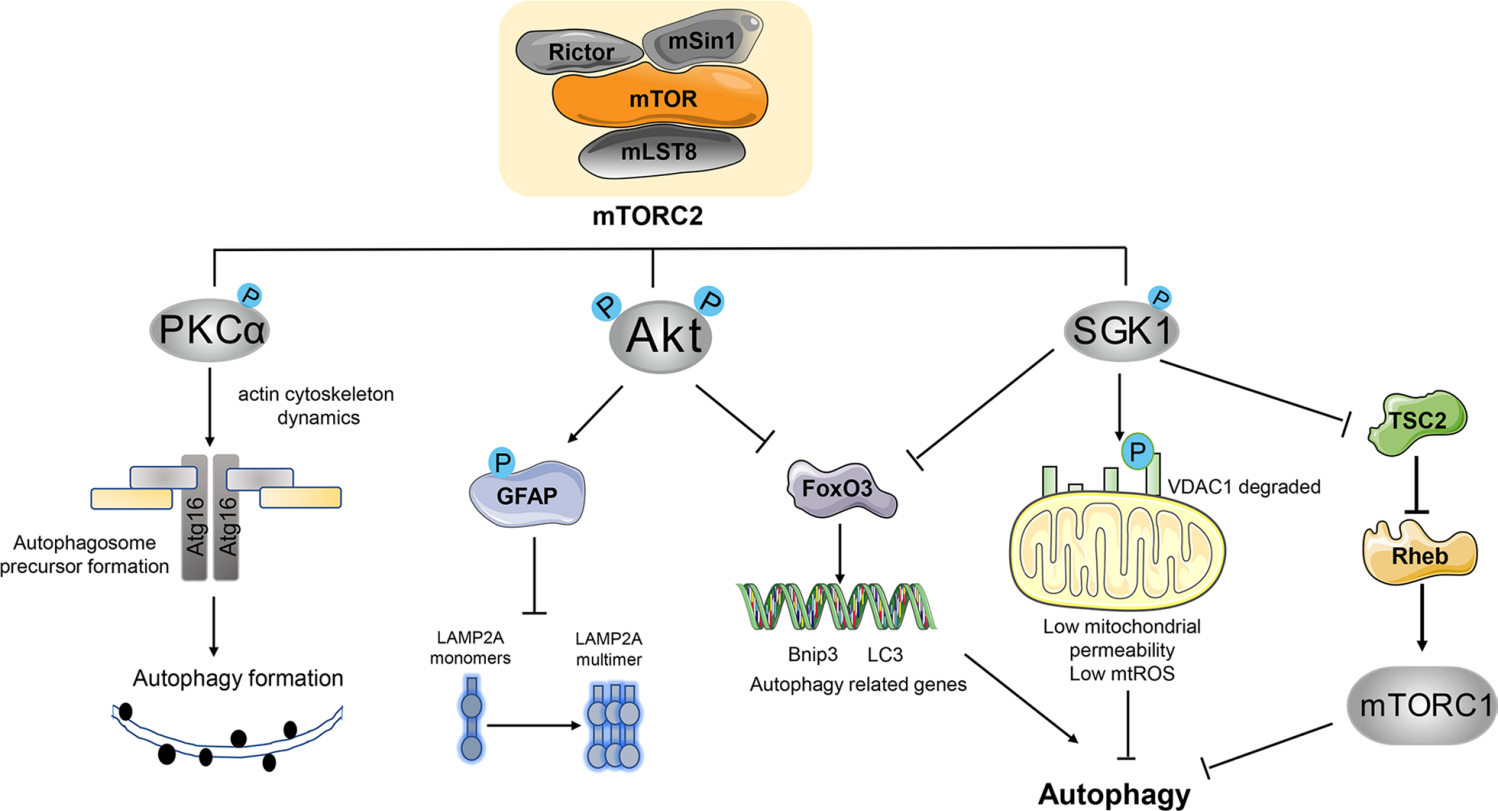
mTORC2 regulates autophagy by phosphorylating AGC kinases (AKT, PKC, and SGK1). The mTORC2/PKC axis promotes autophagosome precursor formation by regulating Atg16; the mTORC2/AKT axis inhibits autophagy by regulating FOXO3 and LAMP-2A; the mTORC2/SGK1 axis inhibits autophagy by regulating mitochondrial homeostasis, FOXO3 and mTORC1

Taken together, mTORC2 functions negatively in both canonical autophagy and CMA through distinct mechanisms (Fig. 3).

### The mTORC2/PKC axis in autophagy

mTORC2 controls cellular cytoskeletal remodeling and cell migration through the phosphorylation of various members of the PKC family, including PKCα, PKCδ, PKCγ, PKCε and PKCζ [27, 28, 39–41]. Autophagosomes are formed from precursor membrane structures (plasma membrane, endoplasmic reticulum and mitochondria) that require autophagy-related proteins, such as the Atg12-Atg5-Atg16 complex [42]. Renna et al. reported that genetic knockdown of RICTOR inactivated PKCα/β, decreased the number of Atg16L1^+^ vesicles, and severely disrupted the actin cytoskeleton [42]. A role for the mTORC2/PKC axis in plasma membrane autophagy has been confirmed. Atg16L1/AP2/clathrin heavy chain interactions contribute to the formation of plasma membrane autophagosomes. The scission of these Atg16L1/clathrin/AP2-associated structures leads to the formation of early endosomal-like intermediates, which is a crucial step in promoting the release and maturation of Atg16L1^+^ vesicles into autophagosomes [43, 44]. Inhibition of mTORC2 reduces the activity of PKCα/β, which then disrupts the actin cytoskeleton, decreases the number of Atg16L1^+^ vesicles, and clathrin-dependent endocytosis. These events lead to reduced autophagosome precursor formation and subsequent inhibition of autophagy [45].

### The mTORC2/SGK-1 axis in autophagy

mTORC2 phosphorylates SGK-1 at S422, thereby facilitating the phosphorylation of PDK1 on residue T265 and resulting in its full activation [46, 47]. mTORC2-mediated SGK-1 activation controls cell survival, as observed in the increased longevity of *Caenorhabditis elegans*, as well as osmoregulation [48, 49]. Activated by both mTORC1 and mTORC2, SGK-1 has been considered to be a negative regulator of autophagy [50, 51]. In murine muscle tissue, genetic disruption of SGK-1 boosts autophagic flux [50]. Studies suggest that SGK-1 functions by phosphorylating and inhibiting the transcriptional activity of FOXO3, which then downregulates ULK1 gene expression to inhibit autophagic flux [52–54].

The mTORC2/SGK1 axis functions as a negative regulator of autophagy through the regulation of mitochondrial homeostasis (Fig. 3). mTORC2 localizes at the mitochondria-associated endoplasmic reticulum membrane (MAM) to maintain membrane integrity and mitochondrial physiology [55]. The translocation of endogenous SGK-1 to the mitochondria has also been observed under cellular stress [56]. Therefore, mTORC2/SGK1 signaling might be potentially involved in mediating autophagy through mitochondrial function. Indeed, recent studies in *C. elegans* suggest an inhibitory role of mTORC2/SGK-1 in autophagy via regulating mitochondrial permeability [7, 57]. *C. elegans* lacking Rictor or SGK-1 showed elevated autophagic flux and shortened lifespan due to increased mitochondrial permeability [7]. It is thought that SGK-1 phosphorylates mitochondrial permeability transition pore (mPTP) component (voltage-dependent anion channel 1) VDAC1 on Ser104, promoting its degradation and maintaining low levels of mitochondrial permeability. This has been confirmed in mice lacking SGK-1, which exhibit exaggerated mPTP-dependent hepatic ischemia/reperfusion injury. These findings suggest that although autophagy is generally considered to be beneficial for longevity, it is harmful in this context [14, 58]. Indeed, these studies indicate that the function of autophagy in longevity is complex and might depend on the modulation of mitochondrial permeability. Consistent with these studies, Aspernig et al. reported that inactivation of mTORC2 and SGK-1 also increased the level of autophagy and autophagic degradation of mitochondria in *C. elegans* [59]. They proposed that mTORC2/SGK1 signaling regulates mitochondrial homeostasis, and that induction of autophagy in mTORC2/SGK-1-deficient animals is triggered by mitochondria-derived reactive oxygen species (mtROS) [59]. An anti-autophagic role of mTORC2/SGK1 has also been observed in mammalian cells. Castel et al. demonstrated that mTORC2/SGK1 signaling sustained mTORC1 activation, and that mTORC1 was the negative regulator of autophagy. Thus, mTORC2 may inhibit autophagy through SGK-1 by activating mTORC1 [60].

Recently, a study suggested that the relationship between mitochondrial function, nutrient signaling and autophagy focused on the regulation of TOR2 in mitochondrial turnover. The mitochondrial prohibitin (PHB) complex is important for mitochondrial morphongenesis and membrane maintenance [61]. They found that PHB depletion suppressed the features which induced by sgk-1 mutants, including impaired mitochondrial homeostasis, lipogenesis and yolk formation. And their data suggested that PHB depletion induced the mitochondrial unfolded protein response, which activated autophagy and probably balanced membrane lipid defects of sgk-1 mutants, extends lifespan. In previous studies, rict-1 and sgk-1 deficiency in C. elegans induce mitochondrial ROS, resulting in mitophagy [57]. Baumeister et al. studied further and shown that TORC2 counteracts autophagy in C. elegans is dependent of TORC2-SGK1-VDAC1 signaling, which maintains mitochondrial function and inhibited mtROS. And this process is independent of other SGK1 target, the FOXO transcription factor DAF-16 and phase II ROS detoxification SKN-1/NFE2L2/NRF2 [62].

The role of the mTORC2/SGK1 axis in autophagy might also depend on distinct environmental stresses [63, 64]. For example, under amino acid deprivation, TORC2 inhibits the Ca^2+^ and Cmd1/calmodulin-dependent phosphatase, calcineurin, through the yeast homolog of mammalian SGK-1 (Ypk1), resulting in the activation of Gcn2 and increased autophagy [63, 64].

Although these studies indicate that the mTORC2/SGK1 axis can function as both a positive and negative regulator of autophagy, the precise mechanisms remain unclear.

## Upstream signaling of mTORC2 on autophagy

The activation of mTORC2 is subject to feedback regulations from multiple upstream and downstream components that spatiotemporally terminate or boost mTORC2 signaling. mTORC2 signaling is also extensively interconnected with other signaling pathways. Thus, mTORC2 might regulate autophagy through multiple feedback and crosstalk mechanisms.

### Upstream regulators of mTORC2

There are several upstream regulators of mTORC2, including nutrient (such as glucose, amino acids, methylglyoxal), intracellular cues (such as small GTPase,) and signals from the plasma membrane [65]. Despite the absence of growth factor receptor signaling mTORC2 can also be activated by glucose via acetyly-CoA-dependent acetylation of RICTOR [66]. The role of amino acids in regulating mTORC2 is depending on cellular content. In multiple human cells, amino acids activated mTORC2 via class I PI3K [67]. In T cell, RICTOR/mTORC2 plays an essential role in amino acids sensing and exert inhibitory effect on mTORC2. In T cells, amino acids may activate the cell clycle by inhibiting mTORC2. Therefor in rict-1 deficient T cells continue to proliferation despite the limiting amino acids [68]. Methylglyoxal is a by-product of glycolysis, and it activates AKT through PI3K/mTORC2 in human colorectal cancer cells [69]. Shin et al.’s study exposited the microbe-derived methylglyoxal effected lifespan by regulating mTORC2 [69]. But preferential activation of the mTORC2 signaling axis by it is still unclear.

mTORC2 also be regulated through intracellular cues, including small GTPase, ROS and so on. Previously, there is a prevailing view that GTPase are downstream effectors of mTORC2. With the further study of mTORC2, researches shown that GTPase also a upstream regulator of mTORC2. mSin1 and RICTOR contains a RasGEFN domain. The characteristic of this domain is N-terminal to the catalytic domain of RAS guanine nucleotide exchange factors. The presence of RasGEFN domain suggests a correlation between mTORC2 and GTPase [70]. Ras family GTPase Rit activated mTORC2 via binding mSin1 when response to ROS [71]. A Rho family Rac1, activated mTORC2 by interacting with mTOR when response to the stimulated of growth factors [72]. ROS can be function as signaling molecules, while high level of ROS can damage cells. In S. cerevisae, TOR/Ypk1 signaling suppresses ROS produced via mitochondria and no-mitochondrial sources. And their data suggested that TORC2/Ypk1 plays an important role in regulating as well as responding to cellular level of ROS [73]. And a recent research shown that DNA polymerase gamma deficiency induced mtROS increasing which promotes RICTOR expression to trigger pro-survival autophagy [74]. In lung cancer cells, glutamine induced the formation of ROS activates mTORC2 by elevates expression of Sestrin2. This process protects cancer cell from glutamine depletion [75]. mTORC2 also be regulated by cell adhesion receptors such as CD146. Xu et al. shown that CD146 interacts with RICTOR in response to growth factor stimulation [76].

### Positive feedback regulation

The insulin/PI3K signaling pathway is upstream of mTORC2. The PH domain of mSin1 is required for the insulin-dependent regulation of mTORC2 activity, which inhibits mTORC2 catalytic activity in the absence of insulin, and activates mTORC2 upon binding to PIP3 [77]. AKT phosphorylates the mTORC2 subunit mSin1 at the T86 residue resulting in activation of mTORC2. Activated mTORC2 then phosphorylates AKT at S473, resulting in a positive feedback loop [78]. This positive feedback loop suggests that partial activation of AKT promotes mTORC2 activation, which then leads to full activation of AKT [78]. Fully activated AKT inhibits autophagy via phosphorylating its effectors, such as FOXO3 to negatively impact CMA [34, 36].

### Negative feedback regulation

mTORC1 functions as a negative feedback regulator that can inhibit mTORC2 activity through either mTORC1-mediated Grb10 phosphorylation or the key effector of mTORC1 (S6K1)-mediated phosphorylation and degradation of insulin receptor substrate1/2 (IRS-1/2), thereby dampening mTORC2 signaling [5, 79, 80]. mTORC2 could regulate autophagy through this complex signaling network.

### Crosstalk with AMPK

AMPK is a regulator of mTORC1, senses cellular energy status by the ration of AMP and ATP. It activated autophagy by phosphorylating ULK1, beginning the process of autophagy AMPK [81–83]. In previous studies, increasing autophagy by AMPK can inhibite the development of heart failure. During energetic stress, mTORC2 can also be activated by AMPK through phosphorylation of S1261 on mTOR [84, 85]. Consistent with these studies, Li et al. suggested that AMPK activation was shown to improve cardiac function in heart failure by attenuating autophagy potentially via mTORC2 activation [85]. In addition, AMPK activates mTORC2 is independently of mTORC1 mediated negative feedback. AMPK directly phosphorylates mTOR and possibly RICTOR to increase mTORC2 activity [65].

### Crosstalk with ROS

Cellular and mitochondrial ROS induce autophagy via activation of mTORC2 in different types of cells. In fibroblasts, cellular ROS-induced mTORC2 activity concomitantly promotes autophagy [86, 87], while in keratinocytes, mTORC2 is a critical link between mtROS and autophagy. Deficiency of DNA polymerase gamma (Polγ) activates mTORC2 through mtROS, and increases autophagy, while knockdown of Rictor or inhibition of mtROS abolishes prosurvival autophagy [88].

## Conclusions

Autophagy is essential during development and cellular homeostasis. Dysregulation of autophagy is closely associated with various diseases, including cancer, aging, neurodegeneration, infection, and cardiovascular disease, many of which have no effective treatments [50, 89–91]. Therefore, further studies aiming to understand the underlying molecular mechanisms regulating autophagy are required for the development of novel therapeutics. The regulation of mTORC2 in autophagy is less understood than that of mTORC1. However, recent studies have suggested that mTORC2 has a differential role in autophagy, shown in Additional file 1: Supplementary Table 1. For example, mTORC2 acts as a negative regulator of autophagy when regulating mitochondrial permeability through the phosphorylation of SGK-1 and provides further evidence of a relationship between autophagy and aging [7, 59]. In addition, mTORC2 can activate AKT, which then inhibits FOXO3 and CMA, as well as activating mTORC1, which also leads to the suppression of autophagy [34, 35, 38]. In contrast, the mTORC2/PKCα/β axis promotes autophagy by increasing the rate of clathrin-dependent endocytosis, which then facilitates autophagosome precursor formation [45].

While mTORC1 has an established role as an inhibitor of autophagy, mTORC2 functions as a multifaceted regulator of autophagy that mediates many biological processes and maintains cellular homeostasis. However, it remains unclear why mTORC2 has evolved such diverse ways of regulating autophagy. To this end, it is helpful to first consider a broad range of upstream inputs that are sensed by and converge on mTORC2, such as environmental stress, starvation, nutritional deprivation, ROS and aging. Each input might have a differential impact on autophagy. Another possible explanation might lie in the downstream effectors of mTORC2. For example, mTORC2 might preferentially activate distinct downstream effectors upon sensing different environmental or cellular stresses. In this context, each effector protein would differentially affect autophagy in a given cell type or a given species. For example, in murine muscle, mTORC2 inhibits autophagy via SGK-1, while in yeast *S. cerevisiae*, TORC2 positively regulates autophagy through Ypk1 [50, 63]. Furthermore, mTORC2 inhibits autophagy through AKT, but promotes autophagy through PKC. In addition, mTORC2 activates AKT and PKC at the plasma membrane and cytoplasm, respectively. Thus, it is possible that cells have evolved such complex regulatory modes for autophagy to cope with diverse environmental or cellular stresses.

As well as understanding the diverse mechanisms of action of TORC2, other interesting and challenging questions remain. As discussed earlier, the regulation of autophagy by mTORC2 is predominantly through its downstream AGC kinase. As mTORC2 is a Ser/Thr kinase, it is possible that mTORC2 might also regulate autophagy by directly phosphorylating autophagy-related proteins of the autophagy machinery. Like many other protein kinases, the activity of mTORC2 relies heavily on its subcellular localization, especially to membranous structures, such as the plasma membrane, mitochondria, endosomes, ER (endoplasmic reticulum), and MAM [92]. Therefore, another question to consider is whether the different subcellular components permit the sensing of mTORC2 to distinct cellular stress or even to trigger organelle-selective autophagy. It is already known that mitochondria-localized mTORC2 plays an essential role in mitophagy [7, 59]. Addressing these questions will greatly extend our knowledge of autophagy regulation, and may lead to the development of potential therapeutics for a broad scope of autophagy-related pathophysiological conditions.

## Supplementary Information

**Additional file 1. Supplementary table 1.** Regulation of mTORC2 on autophagy.

## Data Availability

Not applicable.

## Abbreviations

AKT: Protein kinase B
CMA: Chaperone-mediated autophagy
DEPTOR: DEP domain containing mTOR-interacting protein
DNA-PK: DNA-dependent protein kinase
ER: Endoplasmic reticulum
FOXO1/3a: Forkhead box O1/3a
GFAP: Glial fibrillary acidic protein
GSK3β: Glycogen synthase kinase-3β
Hsc70: Heat shock cognate 71 kDa protein
ILK: Integrin-linked kinase
LAMP2A: Lysosome-associated membrane protein type 2A
MAM: Mitochondria-associated endoplasmic reticulum membrane
mLST8: Mammalian lethal with Sec13 protein 8
mPTP: Mitochondrial permeability transition pore
mSin1: Mammalian stress-activated protein kinase-interacting protein 1
mTOR: Mechanistic target of rapamycin
mTORC1: Mechanistic target of rapamycin complexes 1
mTORC2: Mechanistic target of rapamycin complexes 2
PDK1: Phosphoinositide-dependent protein kinase 1
PHLPP1: Leucine-rich repeat protein phosphatase 1
PI3K: Phosphatidylinositol-3-kinase-related kinase
Rictor: Rapamycin-insensitive companion of mTOR
SGK1: Serum- and glucocorticoid-inducible kinase 1
TSC2: Tuberous sclerosis complex
UPS: Ubiquitin proteasome system
VDAC1: Voltage-dependent anion channel 1
