# Supplementary material for: mTORC2: a multifaceted regulator of autophagy
Source: Cell Commun Signal. 2023 Jan 5;21:4. doi: 10.1186/s12964-022-00859-7 (PMC9814435; doi:10.1186/s12964-022-00859-7)
Supplement: Supplementary file 2 — Additional file 1. Supplementary table 1. Regulation of mTORC2 on autophagy. [file 12964_2022_859_MOESM2_ESM.pdf]

Supplementary table 1. Regulation of mTORC2 on autophagy

Signaling	Effect on autophagy	Mechanism	References
mTORC2-AKT	In fasting muscle, overexpression of activated Akt completely abolished the formation of GFP-LC3-positive [illegible]	In fasting muscle, mTORC2 negatively regulated autophagy by activating AKT which then inhibits FoxO3-Bnip3 pathway	[29]
	Activation of AKT by mTORC2 decreases the level of CMA	mTORC2 inhibits CMA by phosphorylating lysosome-associated AKT and that repress the assembly of LAMP-2A	[29]
	Constitutive activation of FoxO3 leads to mitochondrial degeneration and autophagy	mTORC2 maintains normal level of autophagy by activating AKT and in turn inhibits the activity of FoxO3	[49]
mTORC2/TOR2-SGK1/Ypk1	Lacking SGK-1 resulted in mitochondrial permeability increased; autophagy increased; lifespan decrease	mTORC2 maintains low mitochondrial permeability by phosphorylating SGK-1	[6]
	The autophagic activity of SGK1 knockout mice increased significantly	mTORC2 inhibits autophagy by activating SGK1 and this process may repress the expression of ULK1	[45]
	The autophagic activity increased in mTORC2/SGK-1-deficient animals	mTORC2 contributes to mitochondrial homeostasis and prevents mtROS induced autophagy by activating SGK1	[52]
	Under amino acid starvation, TORC2 is a positive regulator of autophagy	TORC2 inhibits the Ca2+ and Cmd1-dependent phosphatase, calcineurin through Ypk1, to enable the activation of the Gcn2, and promote autophagy	[56]
mTORC2-PKC	IGF-1R depletion inhibits autophagy	In IGF-1R depleted cells, the activation of mTORC2 is inhibited which decreased the activity of PKC, and then damaged autophagosome precursor formation.	[40]

	Blacking PKC can inhibits autophagy	mTORC2-PKCα contributes to kidney fibrosis by increasing autophagic flux	[71]
